# Marigold flower like structured Cu_2_NiSnS_4_ electrode for high energy asymmetric solid state supercapacitors

**DOI:** 10.1038/s41598-020-75879-9

**Published:** 2020-11-05

**Authors:** M. Isacfranklin, R. Yuvakkumar, G. Ravi, S. I. Hong, Foo Shini, M. Thambidurai, Cuong Dang, Dhayalan Velauthapillai

**Affiliations:** 1 Department of Physics, Alagappa University, Karaikudi, Tamil Nadu 630 003 India; 2 Department of Materials Science and Engineering, Chungnam National University, Daejeon, 305-764 South Korea; 3 Centre for OptoElectronics and Biophotonics (COEB), School of Electrical and Electronic Engineering, The Photonics Institute (TPI), Nanyang Technological University, 50 Nanyang Avenue, Singapore, 639798 Singapore; 4 Faculty of Engineering and Science, Western Norway University of Applied Sciences, 5063 Bergen, Norway

**Keywords:** Energy science and technology, Materials science, Physics

## Abstract

The growth in energy devices and the role of supercapacitors are increasingly important in today’s world. Designing an electrode material for supercapacitors using metals that have high performance, superior structure, are eco-friendly, inexpensive and highly abundant is essentially required for commercialization. In this point of view, quaternary chalcogenide Cu_2_NiSnS_4_ with fascinating marigold flower like microstructured electrodes are synthesized using different concentrations of citric acid (0, 0.05 M, 0.1 M and 0.2 M) by employing solvothermal method. The electrode materials physicochemical characteristics are deliberated in detail using the basic characterization techniques. The electrochemical studies revealed better electrochemical performances, in particular, Cu_2_NiSnS_4_@0.1 M-CA electrode revealed high 1029 F/g specific capacitance at 0.5 A/g current density. Further, it retained 78.65% capacity over 5000 cycles. To prove the practical applicability, a full-cell asymmetric solid-state device is fabricated, and it delivered 41.25 Wh/Kg and 750 Wh/Kg energy and power density at 0.5 A/g. The optimum citric acid added Cu_2_NiSnS_4_ electrode is shown to be a promising candidate for supercapacitor applications.

## Introduction

The word nanotechnology now echoes in every corner of the world. The use of nanomaterials is increasing gradually in many fields. The burgeoning modern scientific age has resulted in remarkable prosperity in economics and industrialization. With rapid increase in population, the world is facing severe energy shortage^1,2^. Therefore, industrial and domestic sectors are working together to address the huge demand for energy in the form of electricity. Particularly, significant efforts have been placed in research of energy conversion and development of storage devices^3^. As a result of new ideas, the focus on developing innovative energy storage systems to meet additional energy consumer demand for using innovative gadgets and smart hybrid electric vehicles is increasing^4,5^. Supercapacitors are such energy storage devices that they have high power density than batteries and high energy density than dielectric capacitors. Prominent features of supercapacitors include high durability, fast charging, larger cycling time, low maintenance cost, and long life^6,7^. The performance of a supercapacitor depends on three factors: electrode material, electrolyte, and arrangement of the assembled device. Among these, the electrode material plays significant function in supercapacitor’s task. Electric double layer capacitance (EDLCs) is a phenomenon that saves electricity through double-layer effect. There is no charge transfer, but the charge is induced at its electrode interface as a result of electrostatic induction. On the other hand, pesudocapacitance is a faradaic charge transfer phenomenon that occurs on the surface of the electrodes through faradaic redox, electrosorption, or intercalation processes^8–10^. High surface area and excellent conductivity are the two key features of EDLC-based materials. Commonly used carbon materials such as grapheme, carbon nanotube, activated carbon and carbon fibers are considered as an excellent EDLC material. The main drawback of these materials is that they have relatively low specific capacitance. On contrary, pseudocapacitors have greater specific capacitance due to the occurrence of high charge storage redox reactions on electrodes^11–13^.

Various metal oxides, hydroxides, nitrogen sulfides, and polymers are considered as excellent pseudocapacitive material. Although metal oxides are selected as primary materials for pseudocapacitive behavior, low dielectric potential between the electrode and electrolyte hinders rapid electron transport^14–16^. Nowadays, transition metal chalcogenides (MCs) have been received much attention and interest in creating superior electrode materials in supercapacitor usage because of their exceptional physical, chemical and anisotropic properties. MCs have been used in fuel/solar cells, light emitting diodes, sensors, lithium ion batteries, electrocatalysts and supercapacitors owing to their remarkable properties such as superior life cycle, catalytic activity, better conductivity and low internal resistance. Specifically, MCs exhibits increased electrochemical performances due to their improved mechanical and thermal stability and enhanced electrochemical activity and electronic conductivity. Chalcogenide compounds are excellent in meeting the requirements of various practical applications and have good electrical conduction properties^17–19^. Further, a wide variety of well-synthesized nanostructures play an eminent role in determining electrodes performance. In last decades, mesoporous materials received substantial interest due to their large area and high porosity, which are favored in diverse applications. Numerous methods such as hydrothermal, surfactant-template and sol–gel methods have been used to develop porous nanostructure with large surface area. It is reported that well-synthesized nanostructures are often used to further boost the performances of electrodes^20–23^. This is because good electrochemical reactions are usually found on the surfaces of the first few atomic layers, increasing the capacitance efficiency of electrode materials. The larger surface area found in hierarchical micro/nanostructures further promotes electrochemical reactions. Gao et al. used sodium citrate and thiourea as a solution in SILAR and chemical bath deposition method for thin film fabrications^24^. Here, the role of citric acid has been studied as a structure directing agent in one step solvothermal route, which has a great influence on the formation of the nucleation process. This kind of quaternary chalcogenide materials are widely used in solar cell based applications. Here, we have adapted quaternary chalcogenide Cu_2_NiSnS_4_ for electrochemical energy storage application due to unique property of quaternary chalcogenide materials since it has three metallic cations which contribute more in the electrochemical reaction than the unary metal chalcogenides. Also, the morphological optimization and higher conductivity of the candidate synergistically give better specific capacitance in our work. Comparatively, we have achieved better specific capacitance, stability and practical outcome than the reported literatures by using this quaternary material^25–27^. From these, the major novelty of the work is synthesis method, elemental composition, varying the concentrations of structure directing agent.

## Experimental sections

### Materials

Copper (II) chloride dihydrate (CuCl_2_.2H_2_O; 99% purity), nickel (II) chloride hexahydrate (NiCl_2_.6H_2_O; 99% purity), citric acid (C_6_H_8_O_7_; 99% purity) and thiourea (NH_2_CSNH_2_; 99% purity) were received from Sigma-Aldrich. Tin (IV) chloride pentahydrate (SnCl_4_.5H_2_O; 98% purity) was purchased from Loba Chemie.

### Flower-like Cu_2_NiSnS_4_ microspheres

To understand the effects of citric acid, samples without citric acid (WO-CA) were synthesized by dissolving 0.1 M copper (II) chloride dehydrate, 0.05 M nickel (II) chloride hexahydrate, and 0.05 M tin (IV) chloride pentahydrate in 80 mL de-ionized water and ethanol under constant stirring for 30 min to which a greenish brown solution is produced. To form 0.05-CA solution, 0.05 M molar ratio of citric acid was injected into the above-mentioned solution and stirred for 20 min. Thereafter, 0.4 M thiourea was mixed to the above solution, and the whole mixture was stirred for another 1 h until homogeneous solution is formed. Then, 0.1 M and 0.2 M molar ratios of citric acid were utilized to form other two products. All resultant products were poured into 100 mL stainless autoclaves and the autoclaves were tightly closed to avoid contamination from the environment. Autoclaves were put in furnace and maintained at 180 °C for 16 h. Black precipitates were obtained for all the three samples after they were filtered thoroughly using ethanol, methanol and dried in hot-air oven at 80 °C overnight. Obtained Cu_2_NiSnS_4_ powders were then used for characterization and electrochemical measurements.

### Characterization

Powder X-ray diffractometer (X’Pert Pro; PANalytical) operated at 40 kV and 30 mA with Cukα = 1.5406 Å was utilized. Quality of crystal and interstitial defects was identified via photoluminescence (PL) spectrometer using the Varian Eclipse instrument. To record the vibration modes of Cu_2_NiSnS_4_ microflower, Micro-Laser Raman microscopy (Seiki, Japan) was utilized. Surface morphology of Cu_2_NiSnS_4_ flower-like microstructure was analyzed using the Quanta FEG‐250 SEM instrument at 1 kV electron landing voltage. Cyclic voltammetry (CV) and galvanostatic charge–discharge (GCD) explored electrochemical performance of Cu_2_NiSnS_4_ microflower using an SP-150 workstation (Biologic). Electrochemical impedance spectroscopy (EIS) studies were carried out in the frequency region, 100 kHz–0.1 Hz at 10 mV AC voltage.

### Fabrication of electrode, full-cell and electrochemical measurements

The working electrode was synthesized by nickel foam coating (1 cm × 1 cm with the active material within 2 mg thickness. The SP-150 electrochemical workstation was used to carry out electrochemical measurements. To measure its practical application, Cu_2_NiSnS_4_ microflower electrode was evaluated through the fabrication of an asymmetric full-cell device. To create a full-cell device, 1 M KOH was mixed well in 30 mL DIW with 3 g polyvinyl alcohol (PVA) and maintained at 80 °C for 2 h to make gel electrolytes. To confirm the performance of the electrode material, Cu_2_NiSnS_4_-0.1 M-CA was used as positive electrode (cathode) and the activated carbon as negative electrode (anode). The electrochemical properties of negative anode material were tested within a potential window from 0 V to − 1 V. Whatman filter paper used as separator was sandwiched between the anode and the cathode. The gel electrolyte was pasted on the active material (2 × 2 cm^2^), activated carbon (2 × 2 cm^2^), and the separator paper (2 × 2 cm^2^). Then the anode, separator, and cathode were arranged to make a full-cell device and the device was tightly covered by Teflon tape. The mass balance in two-electrode system was optimized employing $\frac{Q+}{Q-}$ = $\frac{m+ X Csp X \Delta V+}{m-X Csp- X \Delta V-}$. Different scan rates were utilized for this study (10, 30, 50, 80, 100, and 120 mV/s) with a potential range of 0–0.7 V in cyclic voltammetry. Here, the CV curve indicates that the device is having a combination of EDLC battery cell and pseudocapacitive nature. Similarly, various current densities (0.5, 1, 2, 3, and 5A/g), GCD measurements were recorded. Electrochemical impedance spectroscopy was also used to check conductivity measurements in the range of 0.1 Hz–100 kHz. The specific capacitance of prepared Cu_2_NiSnS_4_ electrodes was calculated using following formula:

1 $$C\mathrm{s}=\int \frac{\mathrm{Id}v}{2\times s\times \Delta V\times m}$$

2 $$C\mathrm{s}=\frac{\mathrm{I }. \Delta t}{m.\Delta v}$$

where, ∫ Id*v* is an integral CV curve area, *s* scan rate (mV/s), Δ*V* potential window (V), *m* mass of the product (mg), *I* discharge current (mA), and Δ*t* discharge time (s).

Columbic efficiencies (ɳ%) was obtained from the GCD measurements using the equation as follows:

3 $${\eta \% }= \frac{{t}_{\mathrm{d}}}{{t}_{\mathrm{c}}}\times 100$$

where, *t*_d_ and *t*_c_ are the discharging and charging time.

According to Eqs. (4) and (5), the electrochemical performances of Cu_2_NiSnS_4_//AC solid-state device were evaluated. GCD profile explored at different currents in a potential range of 1.5 V. Further, Cu_2_NiSnS_4_//AC solid-state device energy density (*E*) and power density (*P*) were estimated employing:

4 $$E = \frac{C\mathrm{sp}\times \Delta {\mathrm{V}}^{2}}{2 \times 3.6}$$

5 $$P = \frac{3600 \times \mathrm{ E }}{\Delta \mathrm{t}}$$

where, *E* (W h kg^−1^) represents specific energy, *C* (F g^−1^) denotes ASC specific capacitance, $\Delta \mathrm{V}$ (*V*) the potential window, *P* (W kg^−1^) implies power density, and $\Delta t$ (s) denotes discharge time^28,29^.

## Results and discussion

**Figure 1:**
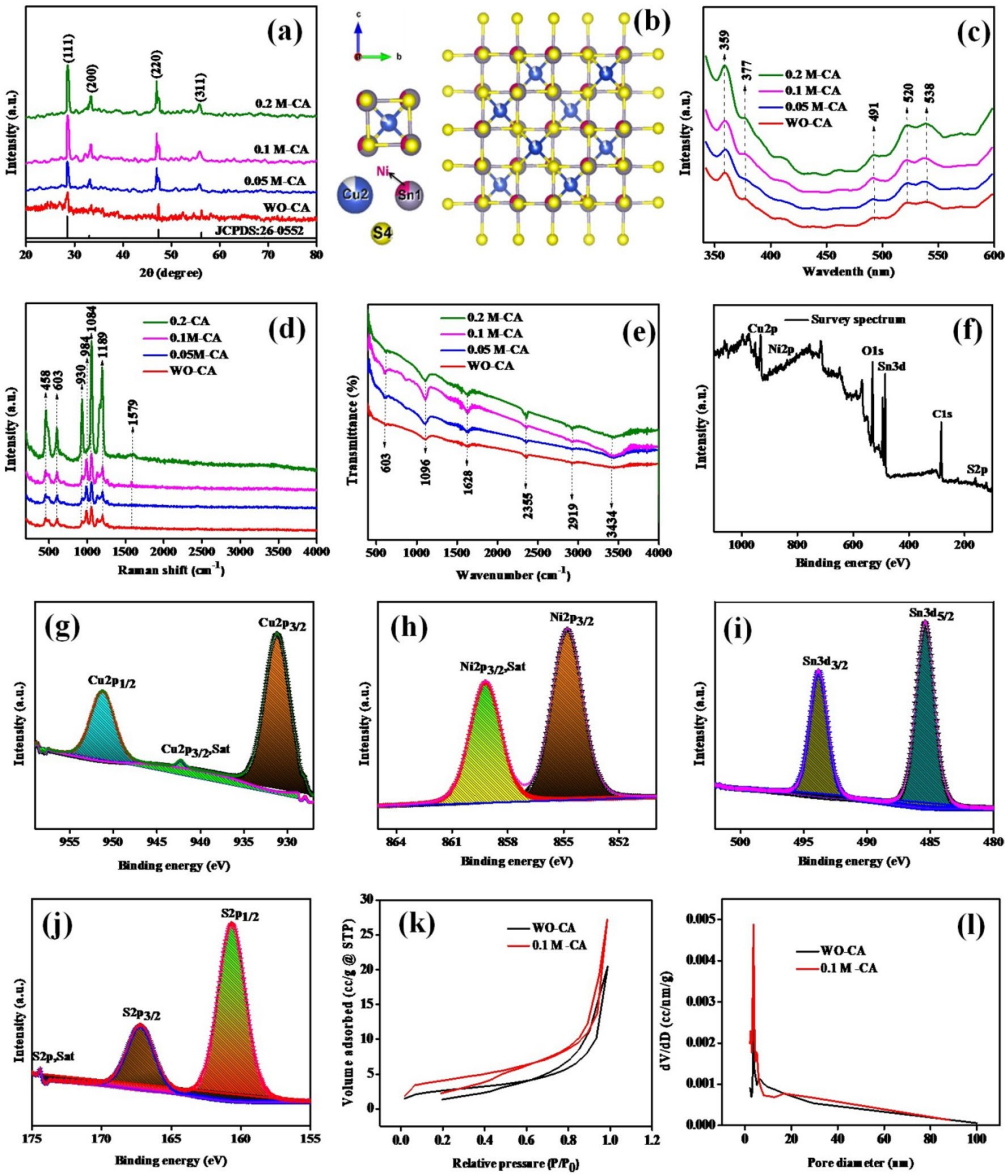
**(a)** XRD, **(b)** ball and stick arrangement of atoms, **(c)** PL, **(d)** Raman, **(e)** FTIR, **(f–j)** XPS spectra, **(k)** BET N_2_ adsorption–desorption isotherm, **(l)** Barrett–Joyner–Halenda (BJH) pore size distribution analysis of Cu_2_NiSnS_4_ without and with citric acid concentration.

Product crystallographic structure and phase purity were identified using the X’Pert Pro PANalytical. Figure 1a shows XRD patterns of the Cu_2_NiSnS_4_ microspheres without and with citric acid, along with their diffracted angles and their corresponding planes. Peaks obtained match perfectly with the standard JCPDS card number 26-0552. The prepared samples do not show any other impurity peaks, indicating a well-synthesized process. All synthesized materials have a cubic structure and space group F-43m (#216-1). The ball-and-stick arrangements of the Cu_2_NiSnS_4_ are shown in Fig. 1b. VESTA software was used to draw the atom arrangements. The radius of the Cu, Ni, Sn, and S atoms are 1.28, 1.25, 1.58, and 1.04 Å, respectively. The photoluminescence (PL) function is based on the mechanism by which the excited electrons generated by the optical excitation and the exited electrons return to ground state by emitting photons. As such, a PL emission spectrum provides information on the crystal defects, recombination of the free charge carriers, oxygen vacancies, and electron trapping, which correspond to the band gap. Figure 1c shows five emission peaks in the wavelength of 359 nm, 377 nm, 491 nm, 520 nm, and 538 nm. PL emission at 359 nm explored Ni^2+^ while peak at 377 nm belongs to near-band emission. Characteristic emission peaks observed at 390–415 nm are responsible for the Ni–S hollow sphere. The peak located at 491 nm could be due to the near-band week green emission; 520 nm explored Cu_2_S. The peaks at 520 and 538 nm are because of interstitial and oxygen-related defects presence. These defects and vacancies provide an active pathway for the transport of ions, thereby improving the electrochemical storage efficiency of the electrode by reducing its diffusion energy at the electrolyte interface^30–32^.

Raman studies are the result of light scattering. During the dispersion process, the molecule vibrates and changes its energy by interacting with it. It also changes the frequency associated with the molecule. It is an effective tool for identifying the vibrational, rotational, and other molecular states of the system. The emission band located at 456 cm^−1^ explored Cu–S stretching vibration, as shown in Fig. 1d. In the synthesized composite, most of the broad luminescence spectrum was observed in the Vis–NIR region within the range of 950–1400 nm. The band observed in the Raman spectrum at 1579 cm^−1^ explored C–C stretching modes. However, some vibrational patterns are missing. Raman vibrations depend heavily on crystal lattice vibrations. While crystal size is reduced to nanoparticles, the vibrational modes of the crystal lattice change to the bulk material. Owing to this reason, Raman vibrations obtained from small nanomaterials tend to be incomplete^33^. Chemical structure variation was studied using Fourier transform infrared (FTIR) spectroscopy. Figure 1e shows the FTIR spectrum of the Cu_2_NiSnS_4_ with different citric acid concentration (0, 0.05 M, 0.1 M. 0.2 M). The obtained 603 cm^−1^ may be due to Ni–S stretching vibration, Sn–S bond, and/or Cu_2_–S stretching; 1096 cm^−1^ is due to asymmetric stretching vibrations. Furthermore, absorption band in the 500–700 cm^−1^ range is typical for Cu–S stretching; 1628 cm^−1^ explored C–C bonding; 2355 cm^−1^ is attributed to atmospheric CO_2_ present in the synthesized product; while the absorption at 2919 cm^−1^ revealed –CH stretching vibrations; 3434 cm^−1^ explored –OH stretching vibrations of intercalated water. Here, both FTIR and Raman techniques confirmed the formation of Cu_2_NiSnS_4_^34,35^.

The Cu2p, Ni2p, Sn3d, and S2p species (Fig. 1f) represent XPS survey spectrum of quaternary chalcogenides Cu_2_NiSnS_4_ composite. Figure 1g displays Cu2p element with two type of spectral lines at binding energy 930.97 eV (Cu2p_3/2_), 942.30 eV (Cu 2p_3/2_, Sat), and 951.10 eV (Cu 2p_1/2_), could be attributed to the Cu^+^ state. No satellite peak was observed for Cu2p_1/2_ at high energies. Ni2p levels observed at 854.80 and 859.18 eV binding energy range are shown in Fig. 1h. Figure 1i shows two peaks at 485.31 and 493.85 eV. The splitting of 8.5 eV indicates Sn^4+^. The element S in the Cu_2_NiSnS_4_ was present at 160.38 eV (S2p_1/2_) and 167.03 eV (S2p_3/2_) (Fig. 1j). An additional *S2p* satellite peak was observed at 174.42 eV^36^.

The Brunauer–Emmett–Teller (BET) studies explored an accurate specific surface area estimation of materials as a function of relative pressure. The N_2_ isotherms of WO-CA and 0.1 M-CA Cu_2_NiSnS_4_ synthesized products were shown in Fig. 1k. Figure 1l shows its corresponding calculated Barrett–Joyner–Halenda (BJH) pore size distributions. According to IUPAC classification, the obtained product exhibit type IV isotherms of mesoporous materials with 3.634 nm and 3.669 nm pore diameter, total pore volume of 0.034 cc/g and 0.045 cc/g and specific surface areas of 11.089 m^2^/g and 15.934 m^2^/g for WO-CA and 0.1 M-CA- Cu_2_NiSnS_4_. Therefore, for 0.1 M-CA-Cu_2_NiSnS_4_ sample, citric acid inevitably increasing contact area due to high interaction of the electrode/electrolyte diffusion of the ions resulting in improved electrochemical performance. The obtained material has a low volume of pores which increases the active area available for absorption or chemical reaction and it offers fast ionic charge transfer in an electrolyte medium^37^.

**Figure 2:**
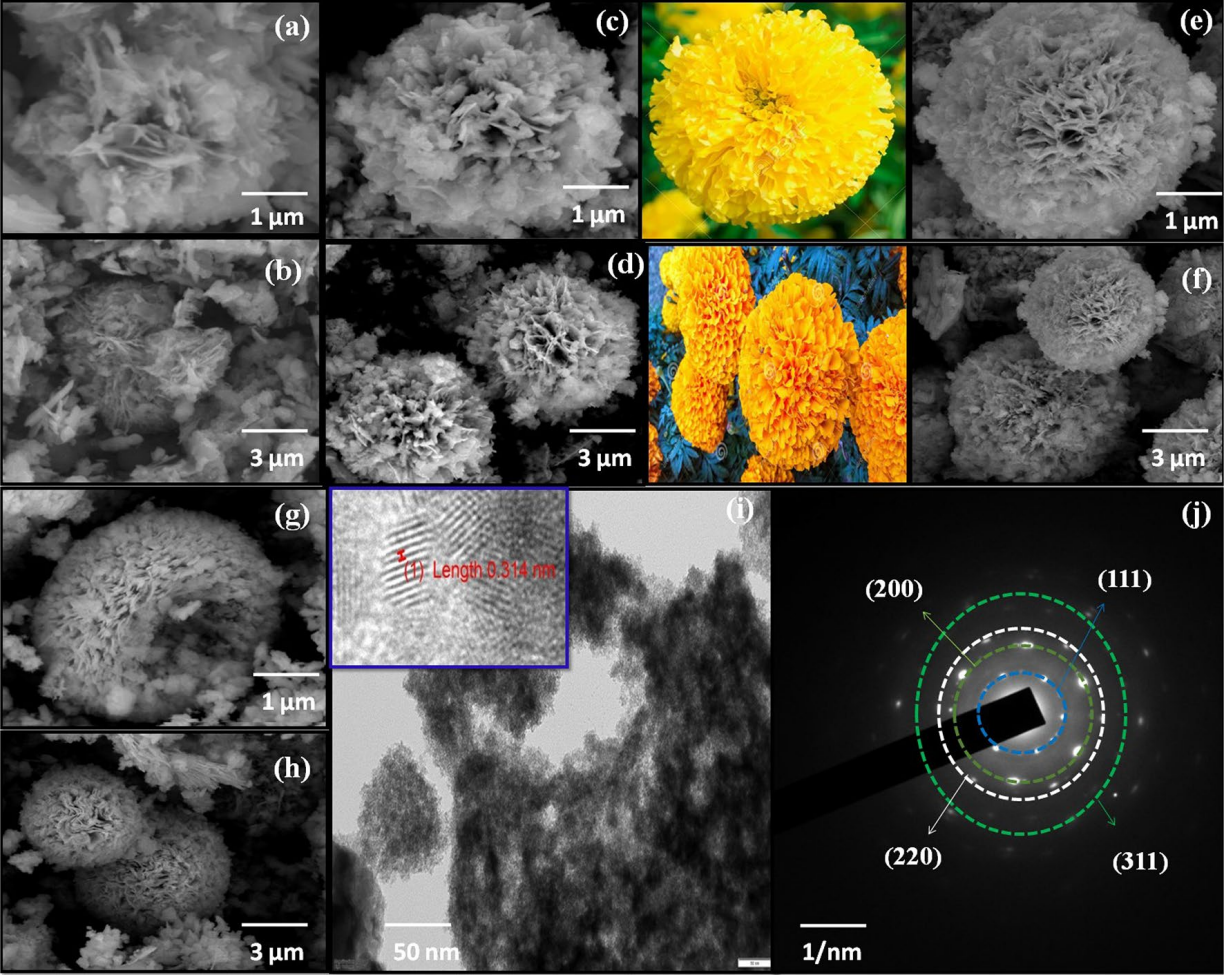
FE-SEM images of Cu_2_NiSnS_4_ **(a,b)** without and with (**c,d)** 0.05 M, **(e,f)** 0.1 M, **(g,h)** 0.2 M citric acid concentration, **(i)** TEM image and **(j)** SAED pattern of Cu_2_NiSnS_4_ with 0.1 M citric acid concentration.

**Figure 3:**
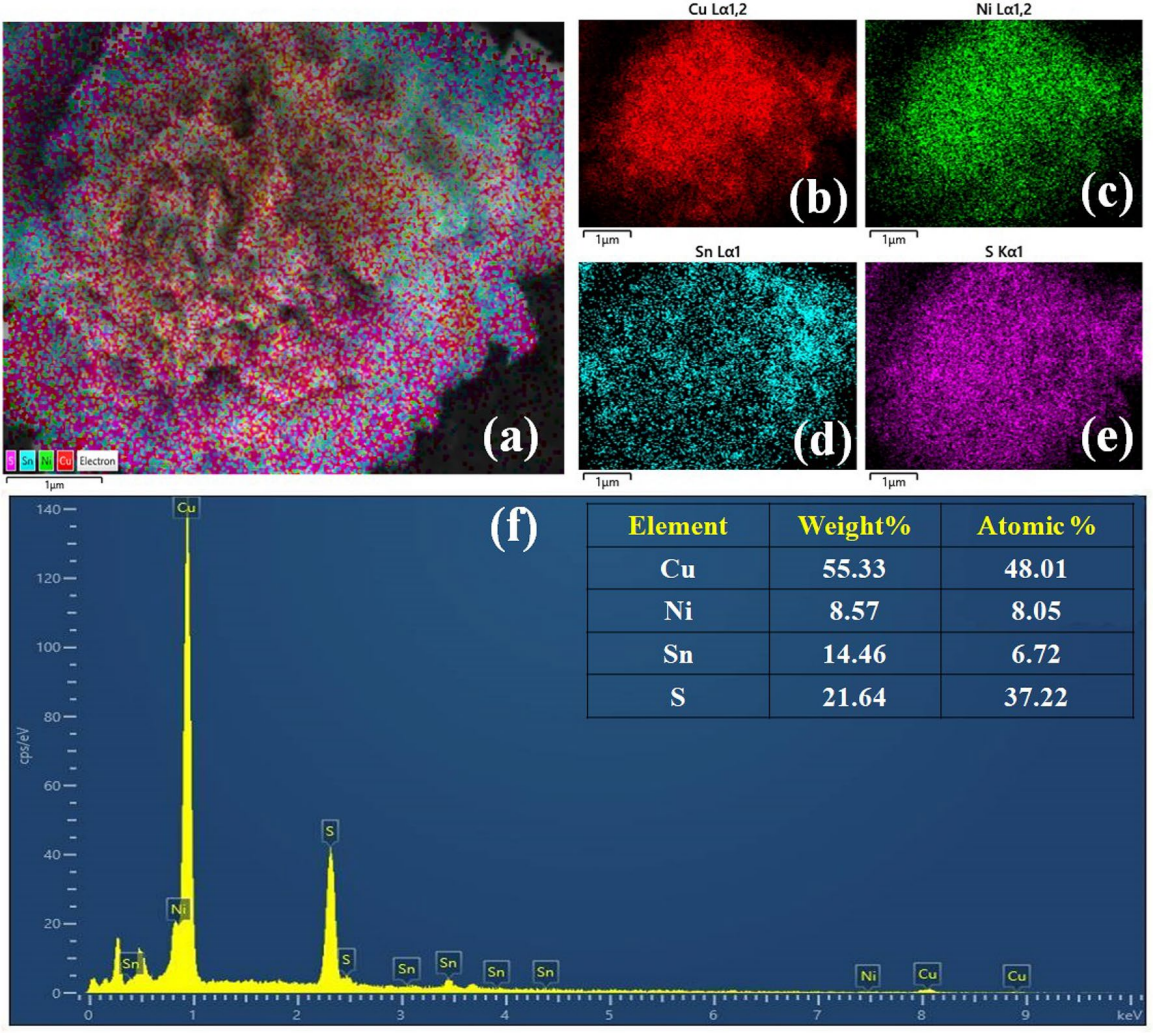
**(a–e)** FE-SEM element mapping, **(f)** EDAX spectrum of 3D-marigold flower-like Cu_2_NiSnS_4_-0.1 M-CA sample.

In solution-based methods, the following parameters determine the kinetic growth: i.e., kinetic energy barrier, temperature, timing, and capping agents. Temperature and time play an eminent role in eliciting changes in the formation mechanism under solvothermal conditions. Flower-like formation mainly depends on the following steps: initial nucleation, aggregation, nascent nuclei, oriented attachment, and Ostwald ripening. Citric acid along with the anionic surfactant such as Cu^2+^, Ni^2+^, Sn^4+^, S^2-^ inevitably reveals flower-like microstructure. Citric acid as structure-directing agent having hydroxyl group and carboxylic groups easily forms complexes with metal ions^38^. Under a constant temperature, increasing the amount of citric acid yields a very good structure (Fig. 2). From FE-SEM images, it is clearly evident that a well-organized microstructure can be obtained in the range of 1–3 µm. Figure 2a,b shows the FE-SEM of WO-CA sample, (c,d) with 0.05 M addition of citric acid on Cu_2_NiSnS_4_, (e,f) with addition of 0.1 M citric acid addition (displays the well-oriented marigold flower like structure as compared with the real flower images), (g,h) shows with addition of 0.2 M citric acid. The petals in the microspheres are in the range of 20–30 nm. The microflower structure favours electrolyte penetration and increasing electrode/electrolyte contact area, resulting in improved electrochemical performance due to the increased electrochemically available active sites^39^. Furthermore, the TEM confirmed presence of microstructures in obtained product (Fig. 2i). A well-resolved lattice fringe (Fig. 2i) confirms 0.31 nm *d* spacing value to most intense peak (111) XRD data. The polycrystalline materials are revealed employing the SAED pattern as shown in Fig. 2j. The SAED patterns further confirmed the other lattice planes in the XRD data^40^. Figure 3a–e shows the EDS mapping of the FE-SEM images. The elemental purity and the element present in the prepared Cu_2_NiSnS_4_-0.1 M-CA sample were examined using Energy-dispersive spectroscopy (EDAX) (Fig. 3f). Cu, Ni, Sn and S are observed with very good atomic and weight percentages. The fact that no other impurity phase found in the synthesized product shows the phase purity and good quality of the sample.

**Figure 4:**
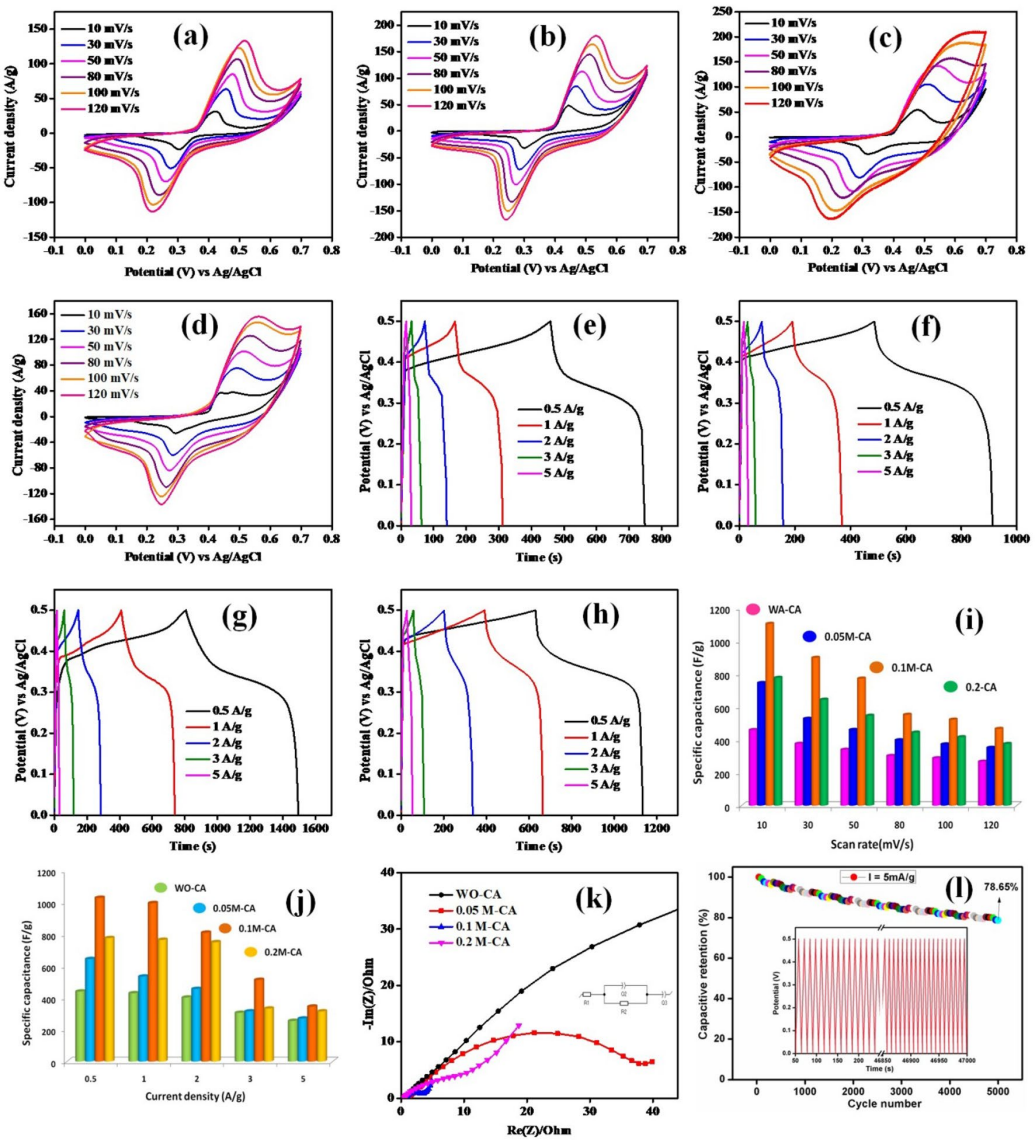
CV cures of **(a)** WO-CA, **(b)** 0.05 M-CA, and **(c)** 0.1 M-CA, **(d)** 0.2 M-CA; GCD curve of **(e)** WO-CA, **(f)** 0.05 M-CA, **(g)** 0.1 M-CA, and **(h)** 0.2 M-CA; cone diagram of **(i)** specific capacitance from CV, **(j)** specific capacitance from GCD, **(k)** EIS spectra and **(l)** capacitive retention of 0.1 M-CA.

Electrochemical performance of the electrode materials was evaluated through electrochemical tools. For studying CV characteristics in three-electrode system, potential rage of 0–0.7 V was maintained for the flower-like Cu_2_NiSnS_4_ over various scan rates, 10–120 mV/s (Fig. 4a-d). 1 M KOH was used as electrolyte, platinum wire as the counter electrode, and Ag/AgCl electrode acted as reference electrode. The redox activity of the Cu_2_NiSnS_4_ electrode was confirmed by the indication of redox peaks couple obtained in CV graph. Here, the redox peak current increases as scan rate increases, as well as shift in oxidation and reduction peaks toward positive and negative potentials observed, which may be due to resistive effect involved with the electrode, polarization, and fast ion kinetics with the increasing scan rate. The increasing current response is proportional to the scan rate, which indicates better capacitive behavior. It could also indicate easy ion diffusion in the electrode material and its large specific area. At high specific capacity low scan rates, the bulk of the active surface is used by the ions for charge storage and hence the result. The well reversible faradaic redox reactions observed even at high scan rates indicate the outstanding electrode material rate capability. The observed redox peaks suggested a linear behavior between current and scan rate in the diffusion-controlled process, where the distribution of oxidation and reduction during OH^−^ diffusion can occur between electrolyte to electrode surface and electrode surface to electrolyte of the medium, respectively. Quaternary chalcogenides increase the contact area of the composite electrode with appropriate selection of Cu_2_NiSnS_4_ microstructure, which increases surface area, conductivity, and porosity and more ions are attracted to electrode surface from which the transport of ions occurs easily. As a result, the conductivity was greatly increased. The specific capacitance of the three different variations of citric acid on Cu_2_NiSnS_4_ was calculated using Eq. (1)^41^. The specific capacitance obtained from CV plots were 458, 375, 339, 301, 287, and 266 F/g for WO-CA; 744, 527, 459, 397, 371, and 350 F/g for 0.05 M-CA; 1104, 897, 771, 551, 522 and 466 F/g for 0.1 M-CA and 775, 643, 545, 443, 415, and 374 F/g for 0.2 M-CA, respectively (Fig. 4a-d).

GCD measurement data was used to calculate specific capacity of the synthesized electrode in the potential range of 0–0.5 V, as shown in Fig. 4e–h. Faradic effects of prepared electrode for different current density values were evident from the nonlinear shape observed with humps in the GCD profile. Equation (2) was used to estimate specific capacitance, which delivered specific capacitance values of 439, 429, 402, 306, and 204 F/g for WO-CA; 643, 534, 456, 315, and 216 F/g for 0.05 M-CA; 1029, 996, 810, 513 and 345 F/g for 0.1 M-CA and 776, 765, 750, 333, and 252 F/g for 0.2 M-CA at different 0.5, 1, 2, 3, and 5A/g current densities. GCD plots show that the discharge time of the 0.1 M-CA is greater than the other variations at both low and high current densities. All the specific capacitance values of CV and GCD are denoted in the cone diagram in Fig. 4i-j. In both CV and GCD, the corresponding specific capacitance values decreased with increasing current density because of low diffusion of electrolyte on high current and incomplete insertion reaction at the available reaction sites, explaining the low specific capacitance values obtained^42^.

Charge transfer ability, conductivity, and the associated resistance of the energy storage electrode at electrode–electrolyte interface were analyzed using standard characteristic electrochemical impedance spectroscopy. Figure 4k shows the Nyquist plot from 0.1 Hz to 100 kHz. A corresponding equivalent circuit was fitted using SP-150 with various capacitance and resistance components. The electrolyte offers the resistance called solution/bulk resistance (*R*_s_), which is initially located in low frequency at Nyquist plot. On the other hand, the high frequency region contains a semicircle part at electrode–electrolyte interface and it has a combination of charge transfer resistance (R_ct_) and the double-layer capacitance (C_dl_) components. Furthermore, Warburg impedance (W) or diffusion resistance is denoted by the presence of a linear line greater than 45˚ in low frequency. R_s_ values of 0.2 M-CA, 0.1 M-CA, 0.05 M-CAand WO-CA was 0.5027, 0.4978, 0.9759, 1.123Ω and those of R_ct_ were 4.969, 1.905, 18.47 and 45.22Ω, respectively. Among these, the sample 0.1 M-CA exhibited very low ionic *R*_s_ and *R*_ct_ values at electrode–electrolyte interface, confirming the high electrical conductivity and fast electron transport kinetics of the Cu_2_NiSnS_4_ microflower in the KOH electrolyte. The higher electron charge transfer may be ascribed to the microflower structure. The excellent electrical contact with low ionic resistance, confirmed from the EIS analysis, indicates that it could be highly suitable for superconductor applications^43^. Durability is the key consideration for practical applications of supercapacitors. The cyclic stability test was conducted to compare the values of specific capacitance to the cycle numbers. At a high current density over 5000 cycles, 78.65% capacity was retained for the best-chosen electrode material, as shown in Fig. 4l. In general, pseudocapacitive products exhibit higher energy density and shorter cycling life at higher current density. This may cause low capacitance retention due to high redox reaction.

**Figure 5:**
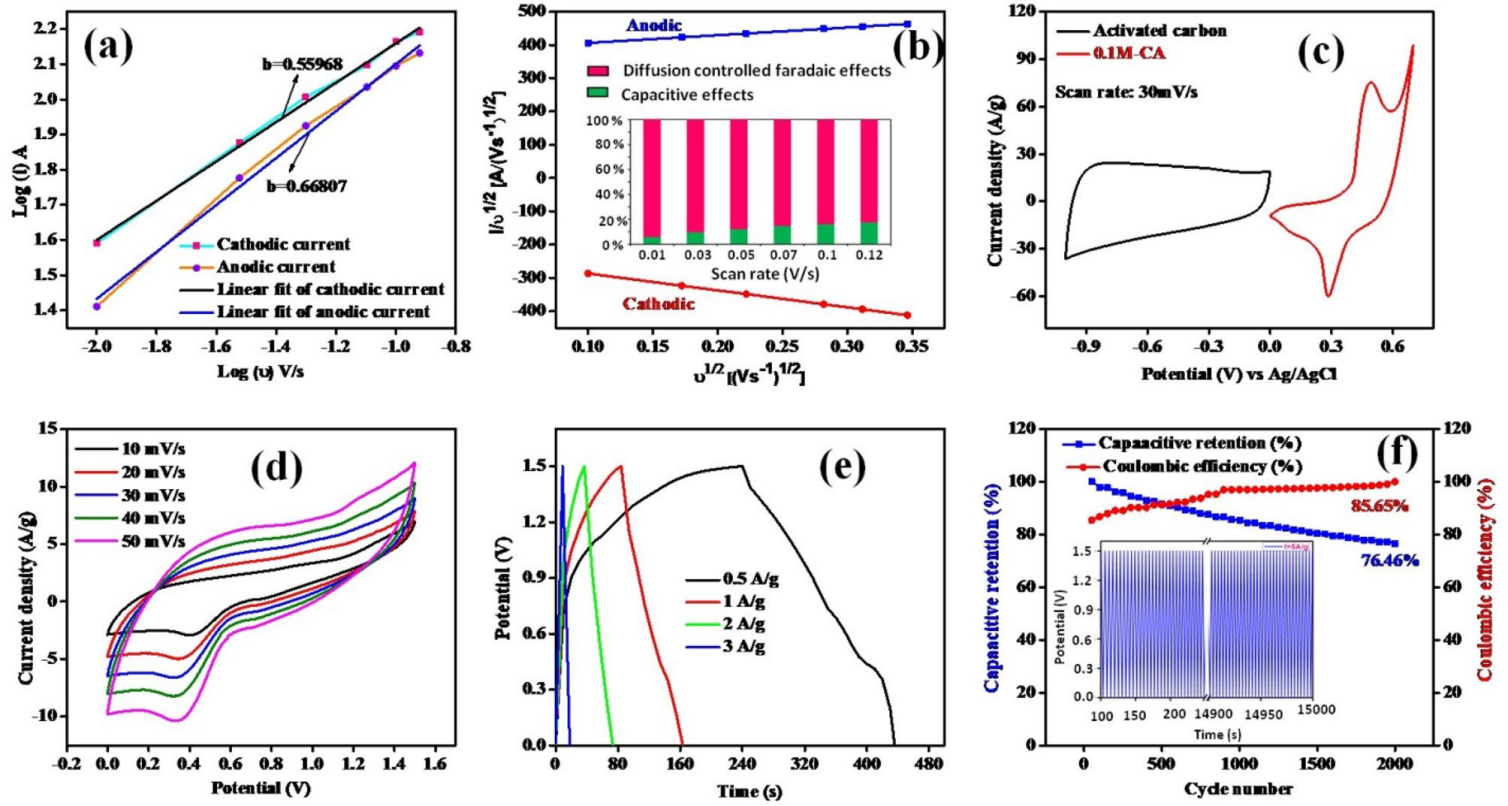
**(a)** log (i) vs log(υ) plot, **(b)** percentage of diffusion-controlled and capacitive-controlled process of Cu_2_NiSnS_4_-0.1 M electrode **(c)** CV curves for 0.1 M-CA and AC at 30 mV/s, **(d)** CV curves of asymmetric supercapacitor, **(e)** GCD curves of asymmetric supercapacitor, **(f)** capacitive retention and coulombic efficiency obtained from two-electrode configuration.

Furthermore, in the CV plot, the total charge stored consists of contributions from three components such as faradaic contribution from electrolyte ions insertion process and charge-transfer process with surface atoms and finally pseudocapacitance and extraordinary contribution from double layer effect. Due to large surface area of the micro/nanostructures, the pseudocapacitance and double layer charging are significant^44^. Capacity effects are characterized by analyzing cyclic voltammetry data at different scan rates using power formula, *i* = *av*^*b*^. The charge storage mechanism is defined by b = 1 that classifies the capacity type charge storage mechanism, and b = 0.5 describes diffusion-limited charge/intercalation behavior of storage mechanism^45^. Figure 5a shows the linear fit graph log (i) vs log (ν) at the redox peak potentials. The linear fit plot gives the b value for 0.55 and 0.66 for cathodic and anodic peak respectively which represents the mixed controlled mechanism of the charge storage electrode. Figure 5b also represents linear fit plot between ν^1/2^ vs i/ν^1/2^ of cathodic and anodic redox peaks for Cu_2_NiSnS_4_-0.1 M-CA electrode. To explicitly calculate the relative contribution of two separate mechanisms, power law i_p_ = k_1_ν + k_2_ν^1/2^ can be modified and this equation could be rearranged into i_p_/ν^1/2^ = k_1_ν^1/2^ + k_2_. Each mechanism of the prepared electrode can be easily identified by calculating both k_1_ and k_2_ variables. The linear fit of I/υ^1/2^ and υ^1/2^ yields slope and intercept values similar to k_1_ and k_2_. Diffusion-controlled contribution was high (94.29%) compared to capacity effects (5.71%) at 10 mV/s scan, and this is clearly evidenced by the current contribution. As scan rate increases, Cu_2_NiSnS_4_-0.1 M-CA diffusion-controlled contribution decreased in small amount. The diffusion controlled and capacitive controlled contribution percentages of different scan rates were displayed in Fig. 5b. However, the diffusion-controlled contribution is much more stable and higher than capacity contribution even at high scan rates, suggesting high capacity of the Cu_2_NiSnS_4_-0.1 M-CA electrode originates mainly from battery behavior^46^.

**Figure 6:**
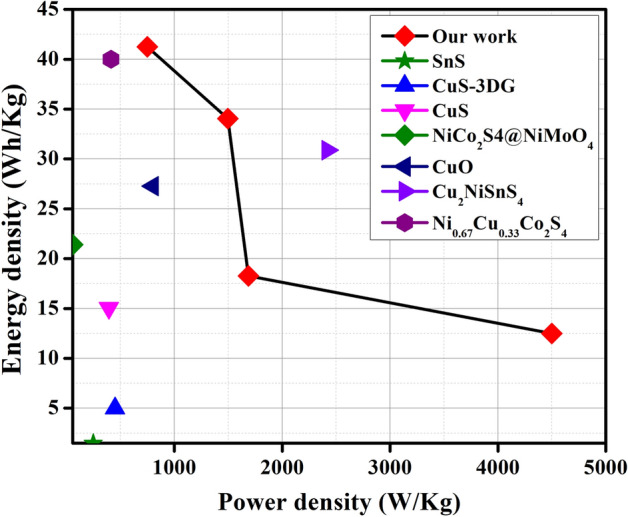
Ragone plot of Cu_2_NiSnS_4_//AC device with some other reported articles.

High specificity and specific power are important factors for designing an asymmetric solid-state supercapacitor device due to its integrated EDLC and pseudocapacitive behavior. To achieve high energy density, in an asymmetric solid-state device marigold flower structure acts as cathode, carbon as anode, Whatman filter pepper as separator, and PVA gel with 1 M KOH as an electrolyte^47^. Figure 5c shows the combined graphs of cathode and anode material, which are in 0–1.5 V. The CV graphs describe the electrochemical performances of electron transfer-initiated chemical reactions, as shown in Fig. 5d. Energy and power density were calculated using Eqs. (4) and (5) from the GCD plot as shown in Fig. 5e. The values of energy and power density are given in Table 1. The maximum energy density (41.25 Wh/kg) and power density (750 W/kg) were obtained at 0.5A/g current density. Figure 5f illustrates the capacitive retention and columbic efficiency of the solid-state supercapacitor. The 76.46% capacity was retained after completing 2000 charge–discharge cycles and 85.65% coulombic efficiency was maintained from the GCD profile. Naturally, the metal sulfide materials are affected by its poor stability. The rapid ion diffusion and fast charge–discharge reaction kinetics were affected when Ni foam used in alkaline electrolyte. The best way to increase stability is to mix carbon based materials (MWCNTs, rGO) with prepared composite. Incorporation of chalcogenides with carbon based nanostructures enhances the electrochemical properties. Furthermore, incorporation of carbon based materials with composite will also increase the stability and capacity. In Fig. 6, Ragone plots of the developed Cu_2_NiSnS_4_/AC device in this study along with other devices reported earlier are depicted and supercapacitive parameters of these devices are listed in Table 2 for comparison.

**Table 1:** Capacitive parameters of energy density and power density from GCD plot.

Potential window (V)	Current (A)	Specific capacitance (F/g)	Discharge time (s)	Energy density (Wh kg^−1^)	Power density (W kg^−1^)
1.5	0.5	132	198	41.25	750
	1	61.5	82	34.06	1495
	2	58.5	39	18.28	1687
	3	37.5	10	12.50	4500

**Table 2:** Literature study for Cu_2_NiSnS_4_ ASS-SC devices that provide supercapacitive parameters.

Materials	Morphology	Specific capacitance@GCD	Energy density (Wh kg^−1^)	Power density (W kg^−1^)	Stability (%)@ cycle	Refs.
SnS	Nanorods	70 F/g@0.5 mA/cm^2^	1.49	248.33	–	^48^
CuS/3DG	Nanoplates	249 F/g@4A/g	5	450	95%@5000	^49^
CuS	Microsphers	237 F/g@0.5A/g	15.06	392.9	88% %@4000	^50^
CuS/C-120@PANI	Nanoparticles	425.53 F/g@1 mA/g	–	–	89.86%@3000	^51^
NiCo_2_S_4_@NiMoO4	Nanotube array	2006 F g-1@5 mA/cm^2^	21.4	58	78%@2000	^52^
CuO	3D flower-shape	612 F/g@1A/g	27.27	800	98%@4000	^53^
Cu_2_NiSnS_4_	Flower-like microspheres	508 F/g@2 A/g	30.88	2.42	95.7%@2000	^37^
Ni_0.67_Cu_0.33_Co_2_S_4_	Spherical cluster nanorods	1340.48 F/g@1A/g	40	412.5	80.45%@10,000	^54^
Cu_2_NiSnS_4_	Marigold flower	1029@0.5A/g	41.25	750	59.31%@5000	This work

**Figure 7:**
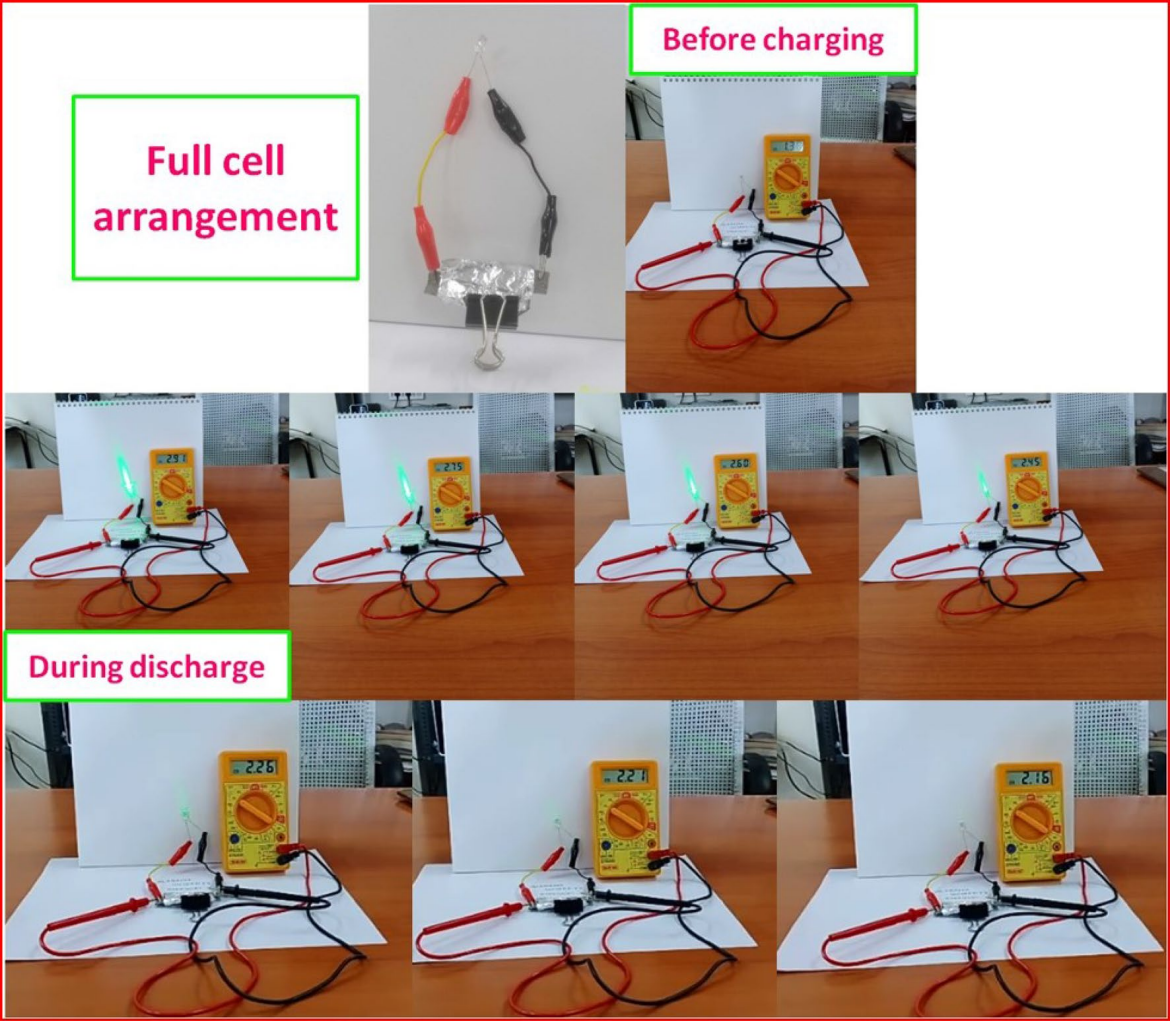
Practical application and observations.

To demonstrate the feasibility of the fabricated electrodes for practical application, solid-state supercapacitor device was prepared in the lab to illuminate a green LED, as seen in the video recording (Supplementary). The working voltage of the green LED has been verified step by step. Various observations of full-cell device before charging and illumination of the LED during discharge are shown in Fig. 7. Generally, the forward voltage of a LED ranges from 1.8 to 3.3 V. It will vary for different colors of LED. Typically, the approximate forward voltage of a green LED is 2.2 V, which means that when the appropriate voltage is applied to LEDs, electrons can be recombined with the electron holes inside device, releasing energy as photons. As illustrated in Fig. 7, a full-cell device before charging shows a voltage of 1.38 V. After charging for 30 s the voltage increased to 2.91 V. When the LED was connected to the supercapcitor device, a decrease in the voltage from 2.91 to 2.16 V was observed during discharge of the device. The low level illumination at 2.21 V confirms the working voltage of the green LED. The LED completely lost its luminosity below 2.16 V as expected for green LEDs. This study clearly shows that the prepared marigold flower-like Cu_2_NiSnS_4_ structure possess high energy density and constitutes a good basis for supercapaitors as energy storage devices.

## Conclusions

The 3D hierarchical Cu_2_NiSnS_4_ microstructures were successfully synthesized by employing solvothermal method. The observed marigold flower-like microstructure greatly enhances the electrode specific surface area, thereby enhancing electrochemical performance of supercapacitor compared to traditional materials reported so far. Supercapacitors made of marigold flower like structured Cu_2_NiSnS_4_ in this study was shown to have high specific capacitance of 1104 F/g at 10 mV/s and 1029 F/g at 0.5A/g from CV and GCD curves, respectively. Capacitive retentions of 78.65% @5000 cycles and 76.46% @2000 cycles from three- and two-electrode configurations were reported. The fabricated solid state device provided 41.25 Wh/kg energy density and 750 W/kg power densities. The luminance voltage of the green LED was demonstrated under laboratory conditions. This study demonstrates the high energy density of supercapcitor based on marigold flower like structured Cu_2_NiSnS_4_ electrode, but there are challenges with the stability that have to be tackled. Stability issue and enhancement of capacitance will be handled by suitable addition of carbon based or polymer materials through the intercalation process in our future work.

## Supplementary information

Supplementary Video 1.
